# Reads2Type: a web application for rapid microbial taxonomy identification

**DOI:** 10.1186/s12859-015-0829-0

**Published:** 2015-11-25

**Authors:** Dhany Saputra, Simon Rasmussen, Mette V. Larsen, Nizar Haddad, Maria Maddalena Sperotto, Frank M. Aarestrup, Ole Lund, Thomas Sicheritz-Pontén

**Affiliations:** Center for Biological Sequence Analysis, Department of Systems Biology, Technical University of Denmark, Kemitorvet, Kgs. Lyngby, DK-2800 Denmark; Bee Research Department, National Centre for Agricultural Research and Extension, P.O. Box 639, Baqa’, 19381 Jordan; National Food Institute, Division for Epidemiology and Microbial Genomics, Technical University of Denmark, Kemitorvet, Kgs. Lyngby, DK-2800 Denmark

**Keywords:** Microbial identification, Marker genes, Whole genome sequencing, Bacterial isolate

## Abstract

**Background:**

Identification of bacteria may be based on sequencing and molecular analysis of a specific locus such as 16S rRNA, or a set of loci such as in multilocus sequence typing. In the near future, healthcare institutions and routine diagnostic microbiology laboratories may need to sequence the entire genome of microbial isolates. Therefore we have developed Reads2Type, a web-based tool for taxonomy identification based on whole bacterial genome sequence data.

**Results:**

Raw sequencing data provided by the user are mapped against a set of marker probes that are derived from currently available bacteria complete genomes. Using a dataset of 1003 whole genome sequenced bacteria from various sequencing platforms, Reads2Type was able to identify the species with 99.5 % accuracy and on the minutes time scale.

**Conclusions:**

In comparison with other tools, Reads2Type offers the advantage of not needing to transfer sequencing files, as the entire computational analysis is done on the computer of whom utilizes the web application. This also prevents data privacy issues to arise. The Reads2Type tool is available at http://www.cbs.dtu.dk/~dhany/reads2type.html.

## Background

Identification of bacteria is important for making accurate clinical diagnoses and for narrowing down the list of potential antibiotics that may be used against the pathogens, and therefore for quickly initiating a medical therapy for treating the patient. In the past, traditional phenotypic and biochemical methods were widely used for bacterial identification [1, 2], as bacterial whole genome sequencing (WGS) was too expensive and difficult to implement. WGS has recently started showing its potential as a cost-effective and rapid solution for medical diagnostics and outbreak prevention. For example, via WGS one can identify species and strain [3, 4] and antibiotic resistance gene [5], as well as make predictions of pathogenicity [6] and identification of novel genes.

The latest development in sequencing technology has contributed to lowering sequencing error, producing longer sequence reads, increasing throughput on modern sequencers, and decreasing sequencing cost [7]. Therefore it is expected that in the nearest future, clinical and industrial microbiological laboratories will have access to their own sequencers. The issue to be faced will then be how to handle and analyze the large amounts of sequencing data to produce useful biological and epidemiological information, for example regarding the identity of pathogens.

The major challenge for taxonomy identification based on sequencing data is the selection of marker genes. The 16S rRNA gene is commonly used for deriving phylogeny and taxonomy of microbes [2, 8], and for bacterial identification in metagenomics samples [9]. This is due to the presence of the 16S rRNA gene in all bacteria, as well as its conserved function [10]. However, the 16S rRNA gene has low discriminatory power at species level for several taxonomic groups [11, 12], for example the Enterobacteriaceae family [11, 13–17]. This lack of accuracy in identifying Enterobacteriaceae species using 16S is due to the high similarity of 16S sequences within the family [18]. To increase the bacterial identification accuracy, one could instead use a combination of several housekeeping genes [19]. However, the larger the marker sequence database is, the slower the bacterial identification process becomes. Therefore, a smaller bacterial marker sequence database needs to be constructed, which contains sufficient data for bacterial identification.

Aligning millions of sequencing reads against tenth of thousands of marker sequences is the second challenge in identifying species based on WGS data. String matching is computationally demanding and the most time-consuming part of the *in-silico* taxonomy identification process. Thus, a strategy to efficiently match two sets of sequences is needed.

A web-based taxonomy identification tool must be able to cope with both an unstable Internet connection (on the client side) and network bottlenecks (on the server side), as file upload failure may halt the analysis before it even starts and too many clients uploading files may cause network issues, respectively. Therefore, to avoid these drawbacks, the development of web-based taxonomy identification should focus on minimizing the amount of data transfer through the Internet.

In this paper we describe the web-application Reads2Type, by which one can rapidly identify the taxonomy of bacterial isolates based on raw WGS data. The user does not need to upload the sequencing data to the server. As far as we are aware, this is the first bacterial identification web server that assigns the computational analysis to the client side, thus avoiding network issues and minimizing data transfer. It was previously shown [20] that Reads2Type performs approximately 2.5 times faster than other tools, given the same taxonomy identification accuracy. This result is based on a benchmark study that compared the performance of the console application of Reads2Type with other tools for taxonomy identification of raw sequencing files. The old version of web-based Reads2Type used Java. However, Java web has limited its features since early 2014, and therefore we rebuilt a web-based Reads2Type on a Node.js environment, which is compiled with Browserify. This enables the version of Reads2Type that we present here to be faster and even more reliable for microbial identification than the old web-based Reads2Type version [20].

## Methods

### Evaluation dataset and computational resources

The evaluation set for selecting Enterobacteriaceae marker genes for the probe database consisted of 30,680 Enterobacteriaceae short read sequencing files from NCBI SRA (Short Read Archive) [21] from 24 different species. To examine the performance of Reads2Type, we evaluated 1003 raw sequencing data of *Campylobacter jejuni*, *Enterococcus faecalis*, *Escherichia fergusonii*, *Escherichia coli*, *Klebsiella pneumoniae*, *Staphylococcus aureus*, and *Salmonella enterica* isolates, sequenced at the Technical University of Denmark. For real-life outbreak data, Reads2Type was tested on 6 publicly available raw sequencing data files from the German 2011 *E. coli* outbreak. In addition, Reads2Type was tested on ERR025475_2, which is one of the *K. pneumoniae* raw read files provided by Sanger (http://www.ebi.ac.uk/ena/data/view/ERR025475). The latter test uses different computational resource (in Amman, Jordan) compared with the former: All computational experiments were conducted on a 2.6 GHz Intel Core i5 CPU, 8 GB memory, Mac OS X Yosemite operating system, except the implementation test on Amman, which was conducted on an Intel Core i7 CPU (@2.20 GHz), 6 GB memory, Windows 7 Home Basic SP-1 64-bit operating system.

### Probe database

A probe database, consisting of fragments of selected marker genes, was used as the reference database for Reads2Type to identify the taxonomy of prokaryotes based on WGS reads. The length of the probe sequences was set to 50 basepairs (bp), as nowadays most sequencing platforms produce reads longer than 50 bp. Moreover, reads with length less than 50 bp have an insufficient proportion of unique sequences that can be mapped to the genome [22].

16S rRNA was used as the main marker gene for the probe database. To increase Reads2Type prediction accuracy, the 16S rRNA sequences for the probe database were predicted from the collection of prokaryotic complete genomes using RNAmmer [23] instead of retrieving 16S rRNA sequences from publicly available databases of targeted sequencing and partial coding sequences. RNAmmer is highly accurate in predicting 16S sequences and may even predict 16S sequences that are not yet submitted to the public RNA databases. The complete genomes of bacteria and archaea were obtained from the NCBI Genome Database in August 26, 2012. This dataset of 2045 different strains consists of 969 different bacterial species, 150 bacterial unspecified species, 105 different archaeal species, and 13 archaeal unspecified species such as genomospecies, endosymbionts, uncultured microbes, and “sp.” organisms (i.e., organisms that have only been typed to the genus but not the species level).

Although 16S rRNA was the main marker gene, DNA gyrase subunit B (*gyrB*) was a better marker gene for Enterobacteriaceae, as shown in the Results section. Hence, the probe sequences were generated by, first, gathering 11,481 16S rRNA sequences, which were predicted by RNAmmer, and 1620 Enterobacteriaceae *gyrB* sequences, which were downloaded from the NCBI nucleotide database. Then, all possible 50 bp fragments were generated from these gathered sequences. The outcome formed the probe database.

### Size reduction of probe database

To improve the performance of Reads2Type, we reduced the size of the probe database via three consecutive steps, which consisted in removing 1) all the 16S rRNA probes that were unique to Enterobacteriaceae, as these have low species identification accuracy, 2) the duplicates that resulted from chopping conserved regions of different strains, and 3) the consecutive probes. To remove the duplicates we followed this procedure: Given a marker gene for each of the 50-mer fragments extracted from this gene, we derived a list of organisms sharing those fragments, and called these fragments probe sequences. Of the 1,268,055 probe sequences that were produced, 1,040,203 were uniquely found in one organism; these are defined as unique probes, and the rest of them were shared between organisms; these are called shared probes. To reduce consecutive probes we retained 50-mers every 25 bp on the marker genes, as a window size of 25 bp is considered dense enough to identify the species of the given isolate.

Via the above mentioned three steps we managed to reduce the size of the probe database down to 61,462, which is ∼20 times smaller than the original size; 40,085 of them are unique probes, the rest are shared probes. The file size of the probe database is 4.6 MB. This database is loaded in the client computer’s memory once the Reads2Type website is accessed by the client.

### Reads2Type

**Fig. 1:**
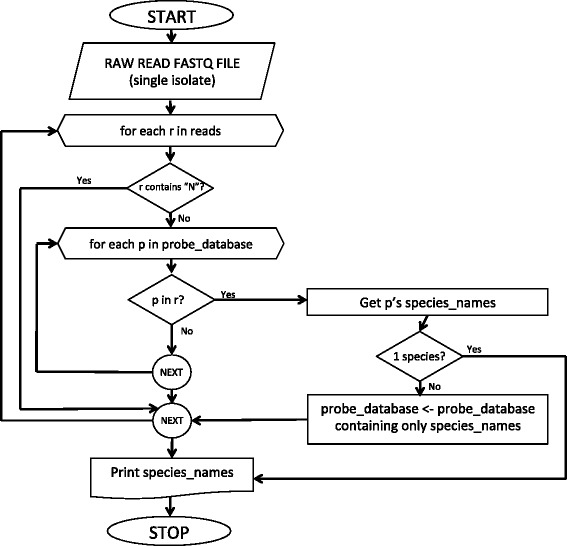
The flowchart of Reads2Type. Each read is aligned with the sequences in the probe database. If a read perfectly matches a shared probe, the probe database undergoes the ‘narrow down’ treatment. Else if it matches a unique probe, the reading terminates and the identified species is presented

Figure 1 illustrates how Reads2Type works. The input of Reads2Type is a raw whole-genome sequencing-file of bacterial isolates. When a read matches a probe sequence, the list of matching organisms is displayed in the user’s browser.

To improve Reads2Type performance, each time a read matches a shared probe, the probe sequences that belong to organisms that are subset of the matching probe are kept, and other sequences are removed, thus significantly accelerating the progress of finding a read that matches a unique probe. For instance, if a read matches a probe sequence that is shared by organisms A, B, and C, then the probe database is reduced in such a way that it only contains the unique probes of A, B, and C, as well as the shared probes of A and B, A and C, and B and C. We define the above as the ‘narrow down’ approach. Also, DNA string matching is done by FM-indexing [24]. This consists in a combination of the Burrows-Wheelers Transform indexing and the suffix array indexing that can be used to efficiently find exact matches to a pattern.

Reads2Type is built on a Node.js environment and compiled with Browserify. Node.js is an open source and multiplatform runtime environment for building server-side and networking applications. Browserify is an open source JavaScript tool that we used to transform Node.js scripts into a client-side web-based compatible script. Once the user chooses the sequencing file, Reads2Type starts identifying the organism’s taxonomy by comparing each read to the sequences in the probe database, and stops running when there is an exact match to a unique probe.

## Results

### The Enterobacteriaceae marker genes choice

**Fig. 2:**
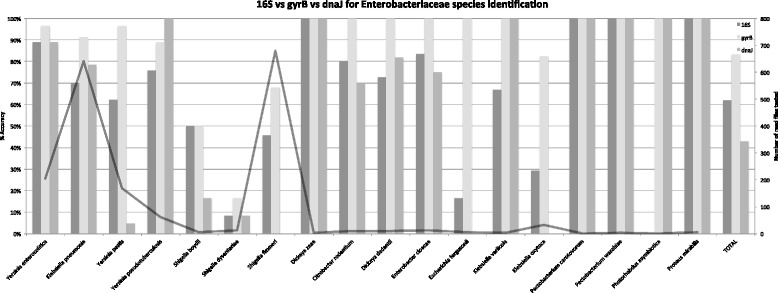
The Enterobacteriaceae species prediction accuracy using 16S, *gyrB*, and *dnaJ* as the marker genes, tested on several SRA read data. For each species, the percent accuracy is shown as three grey bars ranging from 0 to 100 % (*left vertical axis*). The grey line represents the number of read data tested for each Enterobacteriaceae species (*right vertical axis*). The results shown in the TOTAL x-axis tick refer to the accuracy of all tested SRA read data; these data suggest that the *gyrB* marker genes gives a better accuracy than the 16S rRNA and the *dnaJ* marker genes

The prediction accuracy for the three different marker genes 16S rRNA, *gyrB*, and *dnaJ*, which were chosen to identify Enterobacteriaceae species, was compared (Fig. 2). The Enterobacteriaceae species prediction accuracy is higher for the case of *gyrB* genes than 16S and *dnaJ* genes; thus *gyrB* was selected as the marker genes for unique probes. The unique probes of Enterobacteriaceae that are derived from 16S rRNA were subsequently removed, but the shared probes derived from 16S rRNA were kept; this is because using 16S as reference gives less accurate prediction of Enterobactericeae at the species level.

### Reads2Type performance

**Fig. 3:**
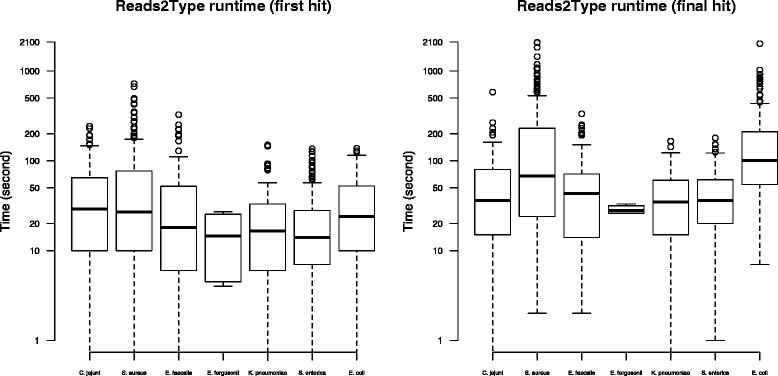
The run time needed to get the first match (*left*) and last match (*right*)

**Fig. 4:**
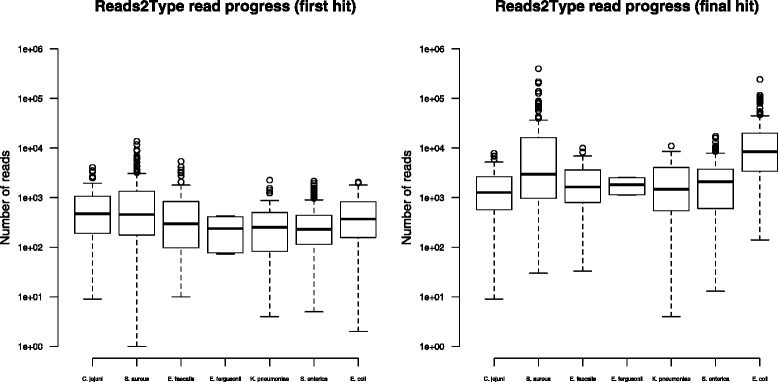
The number of reads required to get the first match (*left*) and last match (*right*)

**Fig. 5:**
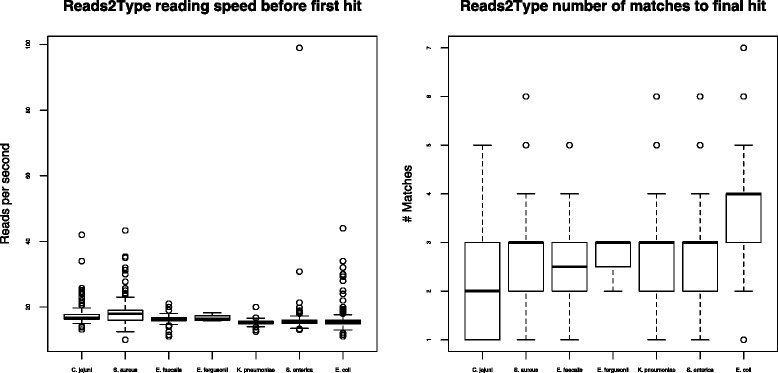
The reading speed before first match (*left*) and the number of matches required to get the final match (*right*)

In the case of in-house raw reads, the Reads2Type bacterial taxonomic identification accuracy is high (Table 1). Only five out of 1003 sequencing files were mistakenly identified. We subsequently discovered that the misprediction of one *S. enterica* raw read file was due to contamination. The other four files, i.e. three *E. coli* and one *S. aureus* raw read files, were misidentified due to 100 % identity of the unique probes that prompt these mispredictions to the draft genomes of the correct species. One should keep in mind that Reads2Type only uses fragments of marker genes derived from complete genomes as the reference database, and therefore this type of misidentification will no longer occur when the complete genome of the true species is available. The total execution time to predict the species varied depending on whether there is a read that matches a unique probe earlier in the sequencing file and whether there are several matches between the reads to shared probes before finally matching the unique probe. The average time needed to get the first match is 40 s (Fig. 3), which is what is required to read about 661 reads (Fig. 4). The reading of the sequencing data progresses faster every time there is a match to a shared probe. Reads2Type reads the sequencing data with a speed of about 17 reads per second (Fig. 5). Most of the Reads2Type runtime is spent on finding the first match to the probe database. When Reads2Type finds a match to a shared probe, reading speed increases dramatically. Therefore, the final bacterial identification is typically reached shortly after the first match (Fig. 3), although the number of reads that needs to be read could reach the order of hundred of thousand (Fig. 4).

**Table 1:** Accuracy test on in-house raw reads

	#True prediction	#False prediction
*Campylobacter jejuni*	107	0
*Staphylococcus aureus*	210	1
*Enterococcus faecalis*	104	0
*Escherichia fergusonii*	4	0
*Klebsiella pneumoniae*	90	0
*Salmonella enterica*	256	1
*Escherichia coli*	232	3
TOTAL	1003	5

Three *E. coli* raw read files were misidentified as one *Shigella dysenteriae* and two *Shigella flexneri*. One *S. aureus* raw read file was misidentified as *Staphylococcus epidermidis*. One *Salmonella enterica* raw reads file was mispredicted as *S. aureus*, which was subsequently discovered to be caused by contamination. Also, two paired-end files were considered as two different files

**Fig. 6:**
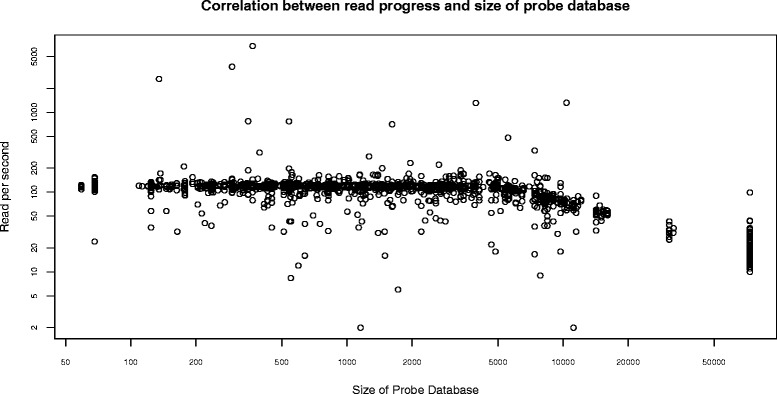
The reading speed as a function of the size of the probe database

Despite having a shorter genome compared with the other six species that we analyzed, on average *S. aureus* needs a longer identification runtime before the first match is achieved. This is because 16S rRNA, which is used to identify staphylococci, is not as good as *hsp60* [25]. Therefore to improve the runtime and accuracy, it is necessary to consider other markers genes than 16S, for example *hsp60*, as we did and discussed in the previous subsection, “The Enterobacteriaceae marker genes choice”. Also, the number of matches needed to identify *E. coli* is the highest among all the seven organisms (Fig. 5). The reading speed as a function of the size of the probe database (Fig. 6) shows that, when the probe database is pruned due to a match with shared probes, the number of probe references is reduced, hence the speed of reading increases.

**Fig. 7:**
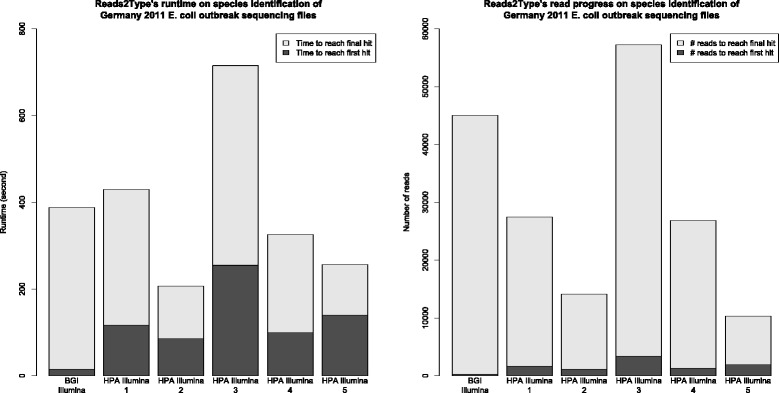
The run time (*left*) and the number of reads aligned before getting a match, called read progress (*right*), which are needed to identify the *E. coli* on the publicly available Germany 2011 *E.coli* outbreak dataset. The dark grey bars and the light grey bars represent the speed of Reads2Type (*left*) and number of reads (*right*) needed to reach the first match and last match, respectively

The runtime and the number of reads (Fig. 7) needed to predict the species of the 2011 German *E. coli* outbreak sequencing data are presented. The result indicates that the runtimes needed to obtain the first match and the last match are consistent with the results shown in Fig. 3 and 4.

To investigate how much a low Internet connection (54.0 Mbps) may affect the bacterial identification process, Reads2Type was tested in Amman (Jordan), which is located on a different continent than where the server is, i.e., Denmark. It turned out that, although the download speed was about 6 times lower than in Denmark, the needed time for bacterial identification in Jordan is similar to the one needed in Denmark (namely less than 10 s), suggesting that the run time is independent from the speed of the Internet on the client side.

**Fig. 8:**
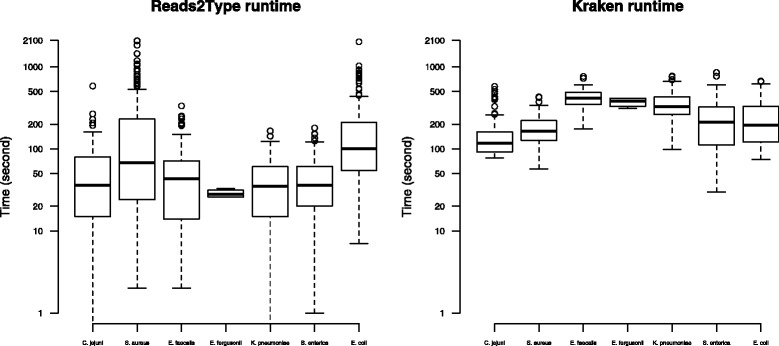
The run time of Reads2Type (*left*), and Kraken (*right*) on the 1003 raw sequencing data

**Fig. 9:**
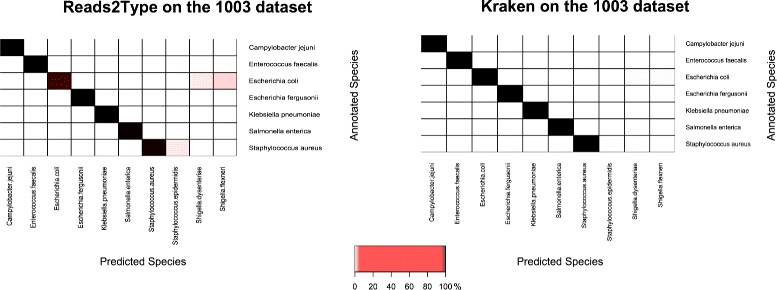
Predictions for seven different species in the 1003 raw sequencing data using Reads2Type (*left*) and Kraken (*right*). For Kraken, the prediction is considered correct if > 50 *%* of the reads are identified as the true taxonomy. Based on this criteria, Kraken has 100 % prediction accuracy. For Reads2Type, the three pink cells correspond to five misidentifications, and the two dark red cells shows that *E. coli* and *S. aureus* has been misidentified, as stated in Table 1

To compare the performance of Reads2Type with that of other tools, we have used Kraken [26], which is a UNIX-based standalone application for taxonomy identification of metagenomic sequence data, and by which one should be able to identify single isolate WGS data. Figure 8 shows that the runtime of Reads2Type generally outperforms the one of Kraken. Figure 9 shows that Kraken is slightly more accurate than Reads2Type when applied on the 1003 raw sequencing data of seven different species.

## Discussion

The results from our study show that by using the web-based Reads2Type application it is possible to identify species based on raw reads of WGS. Also, we show that replacing Enterobacteriaceae’s unique probe sequences, which are derived from 16S rRNA, with the ones that are derived from *gyrB*increases the accuracy of Reads2Type.

Reads2Type is a user friendly web-application that can be accessed via different types of platforms, and which provides species identification on the minutes time scale. This relatively short time scale is due to the ‘narrow down’ treatment (see Section “Reads2Type”), the fast FM index for string matching, the small probe database, and the fact that the computational analysis is performed on the client computers, instead of on the server. In contrast with standalone applications, Reads2Type does not require a downloading and installation of software.

The web-based BLAST tool may be used in alternative to Reads2Type for species identification. Like Reads2Type, BLAST [27] can identify the taxonomy of single isolates based on WGS data. However, BLAST requires conversion of raw sequencing data to FASTA format and uploading of data files to the server. This procedure may be difficult for untrained users. Also, it is time consuming for users that need immediate results or only have a slow Internet connection to their disposal. The accuracy of BLAST is higher than that of Reads2Type, as BLAST uses the nucleotide database, while Reads2Type uses subsets of 16S rRNA sequences and *gyrB* sequences from complete genome and nucleotide database, respectively. However, the web-based BLAST returns an error message when the sequencing file is very large, and as a consequence of this, BLAST may not be able to complete the computation within an hour; while Reads2Type can deal with files of whatever size, as users do not need to upload data files and Reads2Type stops reading the file when the species are identified. The web-based BLAST returns an error, too, if at least hundreds megabytes of high-scoring segment pairs are produced, thus requiring that users readjust the BLAST parameters and re-run the BLAST search.

Short read aligners such as BWA [28] and Bowtie2 [29] may also be used for species identification of a bacterial isolate, although both needs downloading executables. However, both BWA and Bowtie2 require huge computational resources, and these may not be available to a number of users. Although the web-based BLAST and the short read aligners provide accurate results, Reads2Type may be practical when a quick identification of species from raw sequencing files is needed.

To confirm the prediction power of Reads2Type, we used another tool, Kraken. However, running Kraken requires knowledge of UNIX commands and:

1. at least 75 GB of computer memory,
2. at least 160 GB of disk space,
3. a complex and computationally expensive pre-runs, which consist in downloading the installer, running the installation, setting the UNIX environment variables, downloading and prebuilding the reference database from NCBI complete and draft genomes database, and
4. reading the whole sequencing file before delivering results. Thus, Kraken execution time depends on the size of the sequencing file.

In comparison, running Reads2Type needs only limited computer skills and:

1. ∼6 MB of free memory to load the marker database into the browser,
2. no disk space to run,
3. no pre-runs, and
4. no reading of entire sequencing files, as Reads2Type analysis ends when a read matches a unique probe.

One should keep in mind that Reads2Type does not provide prediction confidence as Kraken does. Also, Reads2Type can only be used to identify species of single isolate samples, whereas Kraken can be used for species identification in metagenomic samples. Therefore, if single isolate samples are investigated then one would expect that Kraken predicts only one species with high confidence. Furthermore, the prediction accuracy of Kraken is generally higher than that of Read2Type because Kraken uses both the complete and draft genome sequences as the reference database, while Reads2Type only uses fragments of selected marker gene derived from complete genome sequences.

## Conclusions

The clinical advantage of using web-based WGS tools is that it provides not only a taxonomic identification, but also information regarding antibiotic resistance, virulence factors, novel genes, predictions regarding pathogenicity, and spatiotemporal data concerning previous outbreaks caused by the same or closely related pathogens. This is particularly useful for healthcare personnel that quickly needs to identify the upcoming threat, to help controlling ongoing outbreaks, and to contribute to the development of a global epidemiology map.

In the case when a contamination occurs during the sequencing stage, Reads2Type may misidentify the species. The current availability of complete reference genomes limits the identification power of Reads2Type. This is because the lower the number of strains available as complete genomes is, the less accurate marker probes can be extracted by Reads2Type, hence a lower accuracy in microbial identification follows. However the idea behind Reads2Type is not to substitute the use of accurate tools such as BLAST and BWA, but rather to give an educated guess regarding the identity of bacteria. Furthermore, when using Reads2Type one does not need to be concerned about data privacy issues, as data are not sent across the Internet, and the data analysis is entirely done on the client site.

## Availability of supporting data

The dataset of 1003 whole genome sequenced bacteria were all sequenced at the Technical University of Denmark. The datasets that are already published refers to *S. aureus* [30], *E. faecalis*, and *E. fergusonii* (from project accession [ENA:PRJEB8647]. The remaining raw reads, which refer to *C. jejuni*, *K. pneumoniae*, *S. enterica* and *E. coli*, will be made available upon publication of the papers describing the epidemiology of these strains. Among these, the raw reads of Salmonella-spp-B25, Salmonella-spp-02-03-002, Salmonella-spp-02-03-008, Salmonella-spp-05-102, and Salmonella-spp-07-022 are public [31]. The raw sequencing data files from the 2011 German *E. coli* outbreak were downloaded from ftp://ftp.genomics.org.cn/pub/Ecoli_TY-2482/110601_I238_FCB067HABXX_L3_ESCqslRAADIAAPEI-2_1.fq.gz for the BGI Illumina read data and http://www.hpa-bioinformatics.org.uk/lgp/genomes for the five HPA Illumina read data.
